# Correction: Matha et al. Ergosterol Is Critical for Sporogenesis in *Cryptococcus neoformans*. *J. Fungi* 2024, *10*, 106

**DOI:** 10.3390/jof10080580

**Published:** 2024-08-16

**Authors:** Amber R. Matha, Xiaofeng Xie, Xiaorong Lin

**Affiliations:** Department of Microbiology, University of Georgia, Athens, GA 30602, USA; amber.matha1@uga.edu (A.R.M.); xiaofeng.xie@uga.edu (X.X.)

We recently discovered that the D4H sterol biosensor strains used in Figure 4 of the published paper [1] are not correct, so the authors wish to make the following corrections. For this reason, all prior content regarding D4H strain construction, experimental methods, and experimental data should be removed, namely Figure 4. In addition, panels F and G in Figure 6 should be removed, and the figure (renamed Figure 5 after the removal of Figure 4) should be replaced with the version below. This correction does not change the conclusions drawn by any other experiments performed in this manuscript.

## Text Correction

In the text, a correction has been made to Method’s Section 2.4 Gene Manipulation, the 5th paragraph, except the last statement of this paragraph, should be removed. Method’s Section 2.8 MitoTracker Staining should also be removed. In Methods Section 2.6 Microscopy, mention of D4H in the first paragraph should be removed. The new paragraph should read:

For fluorescence observation of filipin staining, or Dmc1-mCherry, samples were observed with a Zeiss Imager M2 microscope equipped with an AxioCam 506 mono camera. Filipin was visualized with the FL Filter Set 49 DAPI (Carl Zeiss Microscopy, Munich, Germany). The mCherry signal was visualized with the FL filter set 43 HE cy3 (Carl Zeiss Microscopy). All fluorescence images except field of view images were taken with a Zeiss 63x apochromat oil objective with a numerical aperture of 1.4. The field-of-view images were taken with the Zeiss 40x Plan-Neofluar objective lens with a numerical aperture of 0.75.

The Results Section 3.4 D4H Shows Reduced Ergosterol in *sre1*Δ Basidia Compared to Wild-Type Basidia should be removed. Finally, a correction has been made to Results Section 3.6 Overexpression of Multiple Individual EBP Genes Partially Restore *sre1*Δ’s Sporulation Defect paragraph 4 to remove mention of D4H. The new paragraph should read:

With the confirmation of the functionality of the overexpressed *ERG* genes, we proceeded to examine if the overexpression of these genes could rescue *sre1*Δ’s sporulation defect. The overexpression of any of the five *ERG* genes in the wild-type H99 background did not alter sporulation (Figure 5D,E). Interestingly, the overexpression of *ERG2*, *ERG11*, *ERG25*, and *ERG26* partially rescued the sporulation defect of *sre1*Δ in unilateral crosses (Figure 5D,E). Whereas the *sre1*Δ unilateral cross only produced spore chains ~10% of the time, the *ERG2*^OE^*sre1*Δ unilateral cross produced spore chains ~50% of the time, and the *ERG11*^OE^*sre1*Δ produced spore chains ~60% of the time. The *ERG25*^OE^*sre1*Δ and *ERG26*^OE^*sre1*Δ unilateral crosses produced spore chains ~70% of the time (Figure 5E). The additive effect of EBP gene overexpression plus one wild-type copy of *SRE1* in these unilateral crosses (*sre1*Δ x WT) may explain why the sporulation defect was partially rescued during unilateral bisexual mating, but the vegetative growth of these EBP gene overexpression in the haploid *sre1*Δ mutant was not restored on YNB+fluconazole. Taken together, we concluded that ergosterol is a critical nutrient for sporogenesis, and sporogenesis demands heightened expression of EBP genes.

## Figure Correction

Figure 4 should be removed. The figure numbering in the text should reflect the removal of this figure throughout the text. In addition, panels F and G in Figure 6 should be removed and the figure (renamed to Figure 5 after the removal of Figure 4) should be replaced with the version below. This correction does not change the conclusions drawn by any other experiments done in this manuscript.

**Figure 5:**
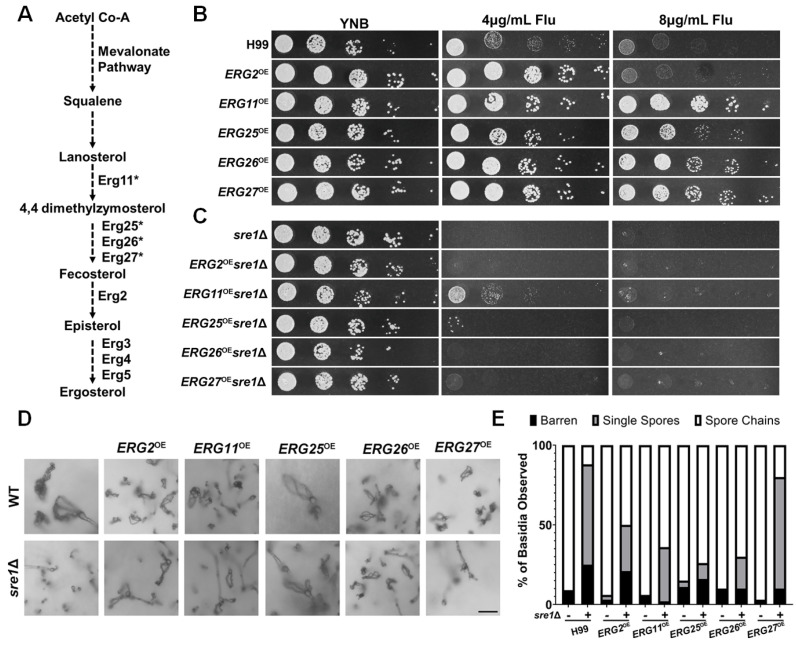
Overexpressed ergosterol biosynthetic genes are functional and can restore successful sporulation in the *sre1*Δ mutant. (**A**) Abbreviated ergosterol biosynthesis pathway highlighting ergosterol genes of relevance for this report. Essential Erg enzymes are marked with an asterisk (*). (**B**) The indicated EBP gene overexpression strains in the H99 were serially diluted and spotted onto YNB and YNB+fluconazole agar media. (**C**) EBP overexpression strains in the *sre1*Δ mutant background were spotted as in panel B. Plates were incubated at 30 °C for two days before imaging. (**D**) The indicated EBP gene overexpression strains with or without the *SRE1* gene were crossed unilaterally with the wildtype strain of the opposite mating type. After two weeks, basidia were visualized to determine sporulation frequencies. Scale bar = 10 µm. (**E**) Frequency of basidia with abnormal spores and spore chains for the crosses tested in Panel A.

## Removal of Citations

The removal of the text described above also requires the removal of the following citations from the original text: 

33. Edwards, P.A.; Ericsson, J. Sterols and isoprenoids: Signaling molecules derived from the cholesterol biosynthetic pathway. *Annu. Rev. Biochem.* **1999**, *68*, 157–185.

36. Marek, M.; Vincenzetti, V.; Martin, S.G. Sterol biosensor reveals LAM-family Ltc1-dependent sterol flow to endosomes upon Arp2/3 inhibition. *J. Cell Biol.* **2020**, *219*, e202001147.

43. Basante-Bedoya, M.A.; Bogliolo, S.; Garcia-Rodas, R.; Zaragoza, O.; Arkowitz, R.A.; Bassilana, M. Two distinct lipid transporters together regulate invasive filamentous growth in the human fungal pathogen *Candida albicans*. *PLoS Genet.* **2022**, *18*, e1010549.

44. Goebels, C.; Thonn, A.; Gonzalez-Hilarion, S.; Rolland, O.; Moyrand, F.; Beilharz, T.H.; Janbon, G. Introns regulate gene expression in *Cryptococcus neoformans* in a Pab2p dependent pathway. *PLoS Genet.* **2013**, *9*, e1003686.

All other citations should be renumbered to reflect the removal of the above citations.

## Acknowledgement Correction

In the original publication we acknowledged Sophie G. Martin from University of Lausanne for the gift of the D4H plasmid. This statement should be removed and the acknowledgements section should now read:

We thank all Lin lab members for their helpful suggestions.

The authors state that the scientific conclusions are unaffected by these changes. This correction was approved by the Academic Editor. The original publication has also been updated. All co-authors have agreed with the content of this correction and wish to apologize for any inconvenience to the readers resulting from this error.

## References

[1] Matha A.R., Xie X., Lin X. (2024). Ergosterol Is Critical for Sporogenesis in *Cryptococcus neoformans*. J. Fungi.

