# Supplementary material for: Ergosterol Is Critical for Sporogenesis in Cryptococcus neoformans
Source: J Fungi (Basel). 2024 Jan 26;10(2):106. doi: 10.3390/jof10020106 (PMC10890046; doi:10.3390/jof10020106)
Supplement: Supplementary file 1 [file jof-10-00106-s001.zip › Supplemental Figures.pdf]

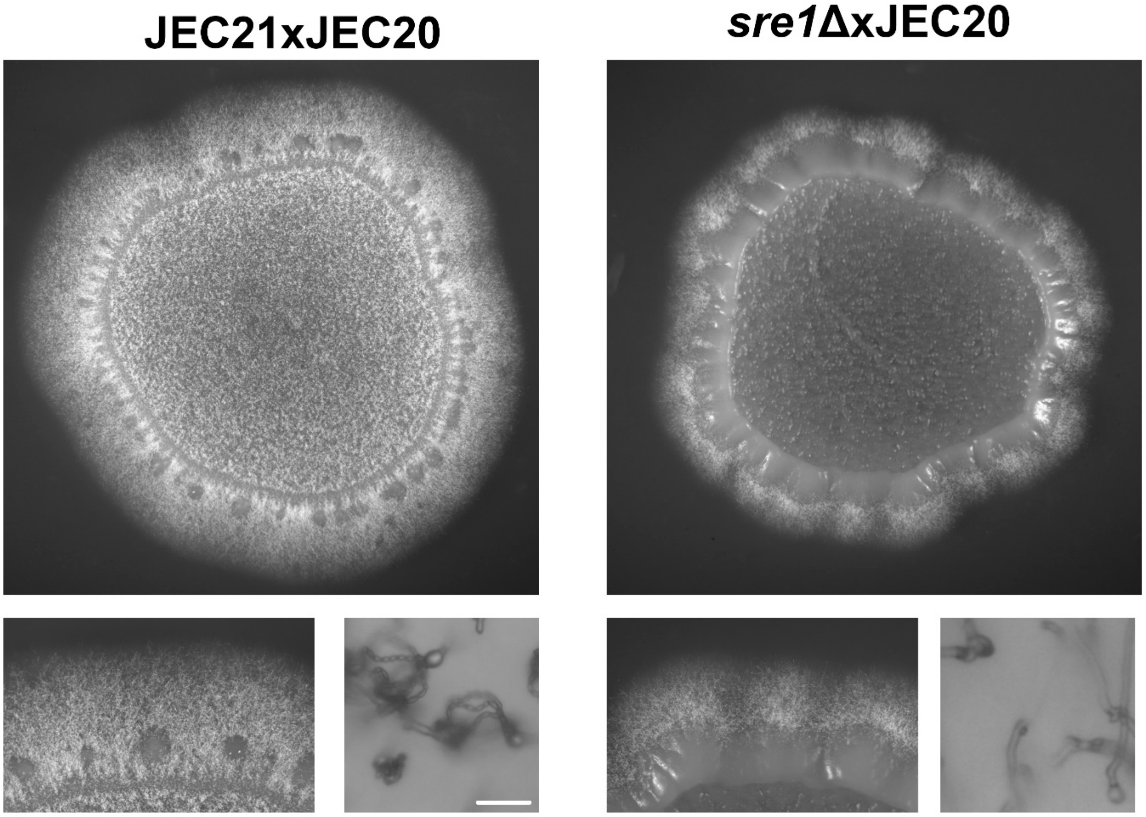

Supplemental Figure S1: A wildtype serotype D cross (JEC21α x JEC20**a**) and a *sre1*Δ unilateral cross (*sre1*Δα x JEC20**a**) were spotted onto V8 pH=7 agar and incubated at 21°C in the dark for two weeks. The whole colony and colony edge were visualized by a stereoscope and basidia were visualized by light microscopy with a 10x objective. Scale bar = 10 μm.

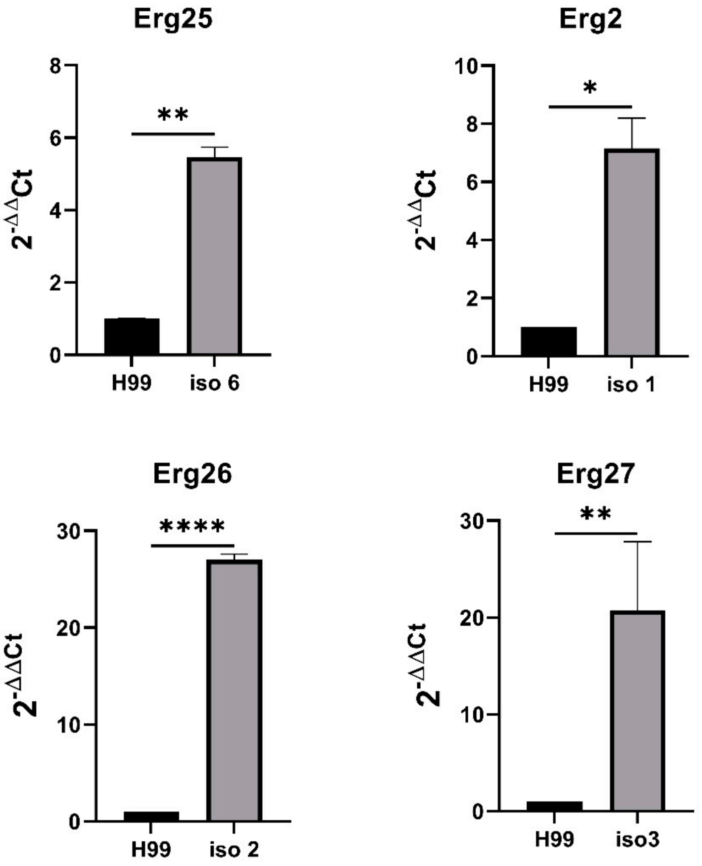

Supplemental Figure S2: RT-PCR data was generated by harvesting cells from overnight YPD cultures. Housekeeping gene *TEF1* was used as an internal control to ensure the quality of the original RNA sample used for cDNA amplification and for normalization. Error bars reflect the standard deviation. Student's t-test was used to assess statistical significance. *: *P*= ≤0.05, **: *P*= ≤0.01, ***: *P*= <0.001, ****: *P*= <0.0001.
